# Prevalence and Intensity of *Pediculus humanus capitis* in Kindergarten and Primary School Children in Poland

**DOI:** 10.3390/jcm14113942

**Published:** 2025-06-03

**Authors:** Marcin Padzik, Gabriela Olędzka, Anita Gromala-Milaniuk, Ewa Kopeć, Edyta Beata Hendiger-Rizo

**Affiliations:** 1 Parasitology Laboratory, Department of Medical Biology, Medical University of Warsaw, Litewska 14/16, 00-575 Warsaw, Poland; gabriela.oledzka@wum.edu.pl (G.O.); edyta.hendiger@wum.edu.pl (E.B.H.-R.); 2 MAMY Z GŁOWY Sp. z.o.o., Raclawicka 29a, 02-601 Warsaw, Poland; gromala-anita@wp.pl (A.G.-M.); ewa.kopec@mamyzglowy.pl (E.K.)

**Keywords:** head lice, pediculosis capitis, infestation, schools, kindergartens, children

## Abstract

**Background/Objectives:** Head lice is an ever-present public health issue, especially among the child population. The diagnosis of head lice infestation should be based on direct examination of the scalp, but, in practice, this standard is often not followed, giving way to indirect methods, such as questionnaires or sales reports of delousing shampoos. In Poland, there is no national pediculosis capitis control strategy; therefore, the aim of this study was to determine the prevalence and associated factors of head lice infestation in schools and kindergartens in the years 2021–2023. **Methods:** Children aged from 3 to 14 years old were directly examined by certified nurses. The same educational institutions were examined across 2021–2023. No personal data of the children were gathered. **Results:** The prevalence of head lice infestation among children ranged from 8% to 13%, with significantly higher intensity and prevalence observed in girls compared to boys. **Conclusions:** The prevalence of pediculosis capitis among children in Poland remains persistent and relatively stable over the monitored period. Gender emerged as a significant factor, showing a strong association with both the intensity and extent of head lice infestation.

## 1. Introduction

The human louse (*Pediculus humanus*) remains a persistent public health concern, particularly among pre-school and school children, due to their direct, host-to-host route of infection [1,2]. There are numerous epidemiological investigations performed worldwide to evidence infestation rates, demographic profiles, and the intensity of affected children’s populations. Most of these studies rely on indirect methods, such as caregiver questionnaires or sales data of anti-lice drugs, which are characterized by a significant methodological bias. Depending on the methodology, population and geographical localization, pediculosis capitis prevalence rates vary considerably. For instance, a survey conducted across 12 primary schools in Norway during 2008–2009 reported a prevalence rate of 41%, solely based on self-observation by pupils/parents, with subsequent periods of the study indicating a decline to 35% [3]. Conversely, a statistical analysis of lice cases confirmed by the Center for Disease Control (CDC) in Iran between 2016 and 2018 revealed an infestation rate of 14.9% [4]. In Honduras, a direct survey of 52 children unveiled an alarming 83% prevalence of lice infestation [5]. In Nigeria, only 0.7% of infestations were detected through direct observation of hair among 1350 primary school pupils [6]. Notably, a study performed in 2019 in Thailand using a cross-sectional survey reported a 50% prevalence of lice infestation among rural women, in contrast to a 3% prevalence among men [7]. In contrast, the study performed in Thailand in 2020 among pre-school and primary school children revealed a prevalence of pediculosis capitis at 68.7% [8].

In Poland, head lice infestations are not subject to sanitary surveillance, meaning that there is no obligation to report cases of pediculosis capitis to the National Sanitary Inspectorate, making it challenging to estimate the scale of the problem. Based on sales data obtained from stationary pharmacies in the years 2019–2023, Dudzinski et al. revealed that increased demand for anti-lice preparations is related to the school calendar and rises when children return to schools after long breaks (holidays) [9]. The level of head lice infestation in school and pre-school children in Poland is difficult to assess due to the lack of comprehensive studies on this topic. In 2023, we published a pioneer study utilizing direct assessment of head lice infestation among over 5000 school and pre-school children, revealing the impact of the COVID-19 pandemic on the decrease in pediculosis capitis prevalence in Poland among school children. The overall pediculosis capitis among examined children varied from 5% up to 12% [10]. Similar results were obtained by other authors. In Israel, a significant drop in sales of pediculicides was observed in 2020, whereas in Argentina, data obtained from the questionnaires showed that the prevalence of lice decreased significantly from before to during the COVID-19 lockdown [11,12]

Prevention against head lice infestation, especially among minors, is very difficult due to poor social hygiene adherence and increased close contact between them. In Poland, screening programs and/or preventive education are not part of any pediculosis capitis control campaigns. Therefore, in our current study, we assessed multiple parameters, including the intensity and extent of pediculosis capitis, the influence of host gender, the type of educational institution, and the caregiver’s educational background on infestation parameters to achieve a thorough understanding of this parasitological concern.

## 2. Materials and Methods

### 2.1. Organization of the Study and Characteristics of the Study Group

A total of 10,943 children were directly examined, comprising 5118 children from kindergartens (pre-school) and 5825 children from primary schools. Children’s examinations in primary schools and kindergartens were conducted by certified nurses. Examinations were performed upon the invitation of the educational institutions. The study was carried out in 53 kindergartens and 30 elementary schools across Poland and lasted from 10 September 2021 to 07 December 2023. Participation of children in the examination was voluntary and performed according to Ministry of Health and Chief Sanitary Inspectorate guidelines. As interpreted by the Chief Sanitary Inspectorate, the parents’ consent to take care of the child at an educational institution is tantamount to agreeing to have the child’s scalp examined (if needed). As a result, parents or legal guardians did not have to provide written consent each time for a head cleanliness check to be carried out on a child.

The studied group consisted of children aged from 3 to 6 years old attending kindergartens and from 7 to 14 years old attending primary schools. The same educational institutions were examined across 2021–2023 in four cities: Warsaw, Poznan, Gdansk and Wroclaw.

### 2.2. Human Head Lice Infestation Verification Methods and Tools

Direct examinations were conducted by certified nurses equipped with specialized magnifying glasses and combs according to the CDC guidelines (leaflet CS249936-E). Each child’s scalp was combed with an individual comb and then carefully examined under a magnifying glass. A group of the same nurses performed all the examinations included in this study. The presence of live lice and/or nits was counted and stratified into the following scale of intensity.
Scale of infestation intensityInfestation intensityDescription0Lack of infestationLack of lice and nits1Mild infestationSingle live louse OR single nits/remains of nits with absence of live lice2Medium infestationFew live lice OR/AND the presence of nits3High infestationNumerous live lice AND the presence of nits

**Figure 1:**
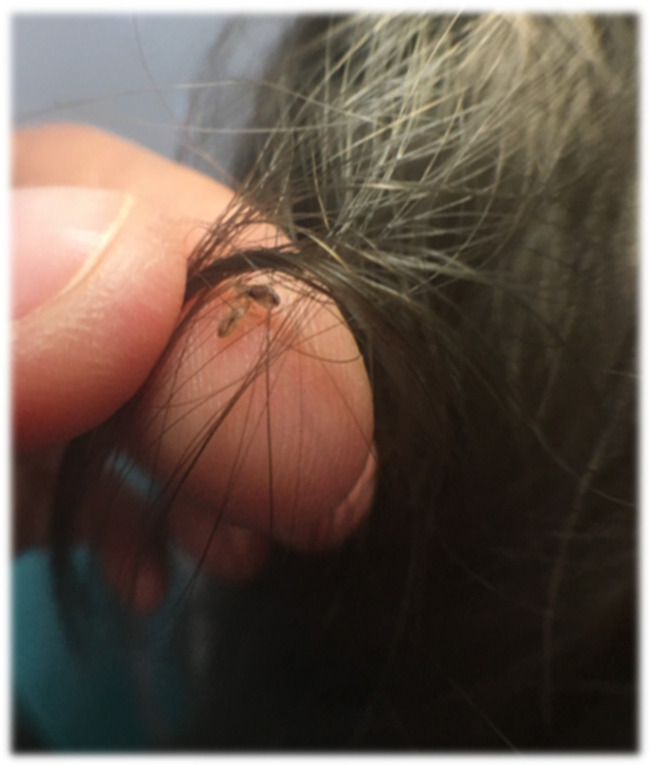
Singe live louse represents level 1 of the infestation.

**Figure 2:**
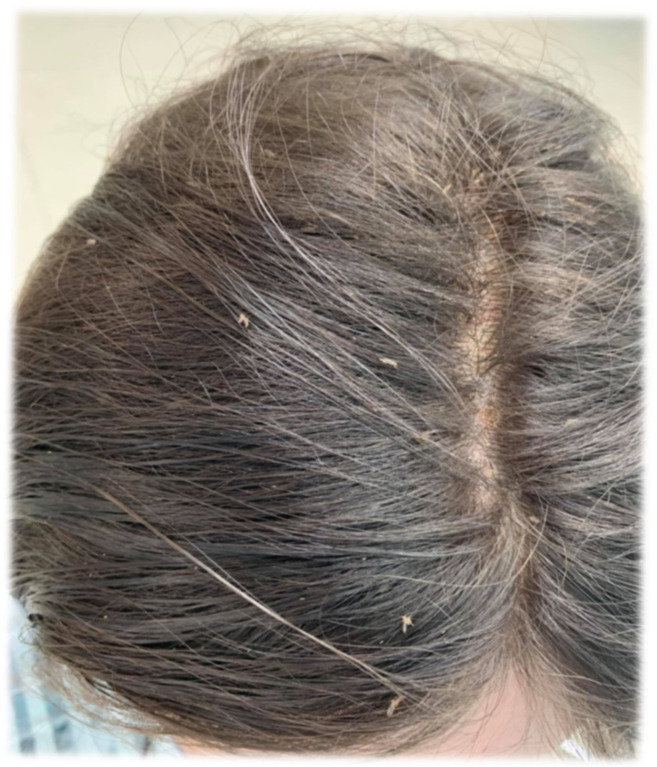
A few live lice represent level 2 of the infestation.

**Figure 3:**
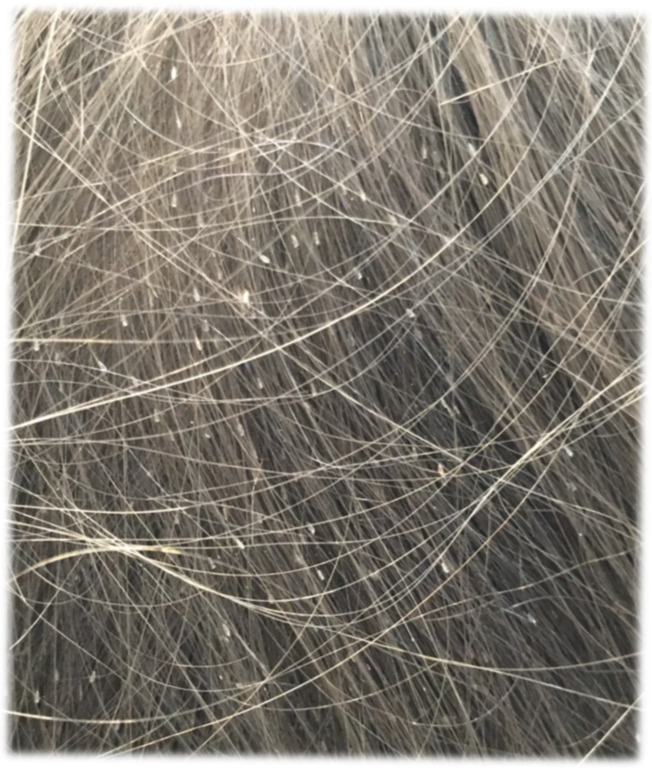
Numerous live lice represent level 3 of the infestation.

Figure 1, Figure 2 and Figure 3 illustrating particular levels of children’s abundance are below.

### 2.3. Statistical Analysis

The statistical analysis was performed using the Fisher Exact Test (socscistatistics.com), accessed on 20 January 2025. All variables were compared with the 2-tailed Fisher’s exact test. The test probability at the level of *p* < 0.05 was considered significant.

## 3. Results

### 3.1. Extensity of Head Lice Infestation

The prevalence of pediculosis capitis remained stable within the school children population throughout the tested years, while a significant decrease was observed in kindergartens when comparing the years 2021 to 2022 and 2023. In 2021 the prevalence of pediculosis capitis was similar when comparing school and kindergarten children’s groups. In 2022 and 2023, the infestation prevalence increased significantly in schools compared to kindergartens. For detailed information and specific data, please refer to Table 1 and Table 2.

Regarding the gender division among children in the year 2021, the prevalence of pediculosis capitis was similar between the genders. In 2022 and 2023, a significantly higher prevalence of pediculosis capitis was found among girls attending both schools and kindergartens compared to boys in the same groups. In 2021 the prevalence of pediculosis capitis was similar between girls in schools and kindergartens and between boys in schools and kindergartens. For girls, we noted the increased prevalence of pediculosis capitis in schools compared to kindergartens between 2022 and 2023. For boys, this trend was only visible in 2023. For detailed information and specific data, please refer to Table 3 and Table 4.

### 3.2. Intensity of Head Lice Infestation

The analysis of pediculosis capitis intensity in schools and kindergartens revealed similar levels of infestation. However, when examining the data on a yearly basis, statistically significant increases in incidence were observed in schools specifically within the mild severity (level 1) of infestation, comparing the year 2021 to the years 2022 and 2023.

In kindergartens, a significant increase in mild intensity (level 1) was noted only between the years 2021 and 2023, while no statistically significant differences were found between the years 2021 and 2022, as well as between the years 2022 and 2023. Moreover, a statistically significant increase in medium-intensity (level 2) incidences was observed in kindergartens between the year 2021 and the years 2022 and 2023.

The results obtained in the years 2022 and 2023 provide evidence that there is a higher percentage of mild and moderate intensity (levels 1 and 2) infestations of pediculosis capitis in the girls’ population compared to the boys’ population. This trend was observed at both educational facilities: schools and kindergartens. For detailed information and specific data, please refer to Table 5, Table 6 and Table 7.

## 4. Discussion

The human louse (*Pediculus humanus*) is a ubiquitous parasitic arthropod afflicting millions of individuals worldwide globally. Despite improved hygiene standards and health awareness, the prevalence of pediculosis capitis continues to be a significant public health challenge [13]. The periodic evaluations of children should be conducted to document the actual head lice infestation prevalence rates. Parents are advised to conduct regular inspections of their children for head lice and administer treatment as required [14]. The aforementioned recommendations for effective head lice infestation control are not followed in Poland, where there is no centralized or mandatory surveillance system for head lice infestation control, which limits the quality, comparability, and continuity of epidemiological data. Consequently, prevalence estimates are frequently derived from indirect sources such as caregiver surveys or sales data of anti-lice treatments, both of which are prone to recall bias and may overestimate or underestimate the true infestation rate.

Transmission occurs primarily via direct head-to-head contact, making pediatric populations more susceptible than adults [15]. Children under 13 years are particularly at risk, and global prevalence estimates vary dramatically, ranging from a few percent to nearly 70%, depending on the country, season, sampling method, and cultural hygiene practices [16,17]. In Poland, reliable national data remain unavailable, largely due to the absence of standardized monitoring. Only a limited number of peer-reviewed studies over the past two decades have addressed the issue, most of which have relied on self-reported infestation or pharmacy sales records. Based on the recent studies performed by other authors, the intensity of head lice infestation in the schoolchildren population varied from 2% up to almost 30% [18,19]. Surveys performed by Bartosik et al. in 2014–2018 reported that 87.5% of the schools in Poland faced incidences of pediculosis capitis, with the greatest number of cases in the group of 6–9-year-olds (68%) [20]. In contrast, data obtained from direct examinations conducted between 1996 and 2000 in the Lubelskie Voivodeship (eastern Poland) revealed that only 0.48–1.59% of school facilities were affected by pediculosis capitis incidences. Children between 8 and 12 years old were most frequently infested, with most cases occurring among girls [21]. Our current direct research showed that the prevalence among school and pre-school children is at a similar level, ranging from 9% to 13% of the tested population. The intensity of infestation was also comparable between schools and kindergartens, with most children experiencing mild infestations. It is noteworthy that in this study only direct examinations of the children’s scalps were performed, so the obtained data are free from bias associated with the use of indirect evaluation methods. Moreover, we analyzed not only school children but also pre-school children attending kindergartens (from 3 years old), which is a unique and commonly not measured dataset.

There are several well-documented factors associated with head lice infestation among children, such as personal hygiene, previous infestation history, household size, socioeconomic status, and gender [22]. As per available literature, gender can significantly influence infestation rates, increasing them by even 3–4 times in girls compared to boys [17]. Studies from Iran, Indonesia, and Ethiopia report prevalence rates ranging from 10.3% to 65.7%. Girls consistently show higher infestation rates than boys [23,24,25,26]. A systematic review and meta-analysis of the data from the past 50 years revealed similarly that the prevalence of pediculosis capitis among boys is significantly lower than among girls (7% compared to 19%) [13]. Data obtained in our study also showed that the infestation rate among girls was approximately twice as high as that among boys in both schools and kindergartens. Furthermore, the findings indicated that the intensity of infestation was also greater among girls, which might be related to the longer hair length observed in this population.

## 5. Conclusions

The results of this study indicate that the prevalence of pediculosis capitis among school and pre-school children in Poland remains persistent and relatively stable over the monitored period. Gender emerged as a significant factor, showing a strong association with both the intensity and extent of head lice infestation. Additional factors—such as personal hygiene, previous infestation history, household size, and socioeconomic status—should be included in future studies to obtain a more comprehensive understanding of the underlying determinants of infestation patterns.

## Figures and Tables

**Table 1:** The prevalence of pediculosis capitis among the tested years within schools and within kindergarten groups, without considering gender division. Statistically significant *p*-values (*p* < 0.05), indicating significant differences between specific years, are bolded.

Years	Schools			Kindergartens		
	Children Infested	Children Clean	Percent of Infested Children	Children Infested	Children Clean	Percent of Infested Children
2021	41	377	9.80	38	262	12.66
2022	162	1183	12.04	166	1727	8.77
2023	427	2928	12.72	328	3304	9.03
Years	*p*-Value					
2021/22	0.2933			**0.0413**		
2022/23	0.5945			0.7658		
2021/23	0.1343			**0.0486**		

**Table 2:** The prevalence of pediculosis capitis between schools and kindergartens among the tested years, without considering gender division. Statistically significant *p*-values (*p* < 0.05), indicating significant differences between specific years, are bolded.

Years	Percent of Infested Children in Schools	Percent of Infested Children in Kindergartens	*p*-Value
2021	9.80	12.66	0.2295
2022	12.04	8.77	**0.0026**
2023	12.72	9.03	**<0.00001**

**Table 3:** The prevalence of pediculosis capitis among the tested years within schools and within kindergarten groups, considering gender division. Statistically significant *p*-values (*p* < 0.05), indicating significant differences between specific years, are bolded.

Years	Schools		Kindergartens	
	Percent of Infested Girls	Percent of Infested Boys	Percent of Infested Girls	Percent of Infested Boys
2021	11.61%	7.73%	13.73%	11.56%
2022	16.31%	6.07%	11.90%	5.07%
2023	15.97%	8.78%	12.73%	4.32%
Years	*p*-Value			
2021/22	0.253		0.7309	
2022/23	**<0.00001**		**<0.00001**	
2021/23	**<0.00001**		**<0.00001**	

**Table 4:** The prevalence of pediculosis capitis between schools and kindergartens among the tested years, considering gender division. Statistically significant *p*-values (*p* < 0.05), indicating significant differences between specific years, are bolded.

Years	Percent of Infested Girls			Percent of Infested Boys		
	Schools	Kindergartens	*p*-Value	Schools	Kindergartens	*p*-Value
2021	11.61%	13.73%	0.6342	8.38%	13.08%	0.2626
2022	16.31%	11.90%	**0.0074**	6.46%	5.34%	0.4744
2023	15.97%	12.73%	**0.0043**	9.63%	4.52%	**<0.00001**

**Table 5:** The abundance of pediculosis capitis within the tested years in schools and kindergartens, without considering gender division. Statistically significant *p*-values (*p* < 0.05), indicating significant differences between specific years, are bolded.

Years	The Abundance of Infestation in Schools				The Abundance of Infestation in Kindergartens			
	0	1	2	3	0	1	2	3
2021	90.19%	5.02%	4.55%	0.24%	87.33%	3.33%	9.00%	0.33%
2022	87.96%	8.33%	3.64%	0.07%	91.23%	6.08%	2.64%	0.05%
2023	87.27%	9.27%	3.22%	0.24%	90.97%	6.44%	2.48%	0.11%
Years	*p*-Value							
2021/22	0.2933	**0.0422**	0.4681	0.4185	**0.0413**	0.0782	**0.0001**	0.2555
2022/23	0.5945	0.3683	0.4741	0.4606	0.7658	0.6419	0.7191	0.6664
2021/23	0.1343	**0.0054**	0.1958	1	**0.0486**	**0.0431**	**0.0001**	0.3283

**Table 6:** The abundance of pediculosis capitis within the tested years in schools compared based on gender division. Statistically significant *p*-values (*p* < 0.05), indicating significant differences between genders, are bolded.

	The Abundance of Infestation in 2021				The Abundance of Infestation in 2022				The Abundance of Infestation in 2023			
	0	1	2	3	0	1	2	3	0	1	2	3
Girls	88.39%	5.80%	5.36%	0.45%	83.69%	11.08%	5.22%	0.00%	84.03%	11.95%	3.69%	0.33%
Boys	92.27%	4.12%	3.61%	0.00%	93.93%	4.46%	1.43%	0.18%	91.22%	6.01%	2.64%	0.13%
*p*-value	0.7763	0.5075	0.4852	1	0.1588	**0.0001**	**0.0003**	0.4168	0.11	**<0.00001**	0.1155	0.3074

**Table 7:** The abundance of pediculosis capitis within the tested years in kindergartens based on gender division. Statistically significant *p*-values (*p* < 0.05), indicating significant differences between genders, are bolded.

	The Abundance of Infestation in 2021				The Abundance of Infestation in 2022				The Abundance of Infestation in 2023			
	0	1	2	3	0	1	2	3	0	1	2	3
Girls	86.27%	5.23%	8.50%	0.00%	88.10%	8.20%	3.61%	0.10%	87.27%	8.94%	3.59%	0.20%
Boys	88.44%	1.36%	9.52%	0.68%	94.93%	3.57%	1.50%	0.00%	95.68%	3.26%	1.06%	0.00%
*p*-value	0.9326	0.1063	0.8422	0.4917	0.2712	**0.0001**	**0.0059**	1	0.0595	**<0.00001**	**<0.00001**	0.1359

## Data Availability

The datasets used and/or analyzed during the current study are available from the corresponding author on reasonable request.

## References

[1] Hill N. (2006). Control of head lice: Past, present and future. Expert Rev. Anti. Infect. Ther..

[2] Nolt D., Moore S., Yan A.C., Melnick L. (2022). Committee on infectious diseases, committee on practice and ambulatory medicine, section on dermatology. Head Lice Pediatr..

[3] Birkemoe T., Lindstedt H.H., Ottesen P., Soleng A., Næss Ø., Rukke B.A. (2016). Head lice predictors and infestation dynamics among primary school children in Norway. Fam. Pract..

[4] Adham D., Moradi-Asl E., Abazari M., Saghafipour A., Alizadeh P. (2020). Forecasting head lice (*Pediculidae: Pediculus humanus capitis*) infestation incidence hotspots based on spatial correlation analysis in Northwest Iran. Vet. World.

[5] Jamani S., Rodríguez C., Rueda M.M., Matamoros G., Canales M., Bearman G., Stevens M., Sanchez A. (2019). Head lice infestations in rural Honduras: The need for an integrated approach to control neglected tropical diseases. Int. J. Dermatol..

[6] Okoh B.A., Alikor E.A. (2013). Prevalence of head lice infestation in primary school children in Port Harcourt. East Afr. Med. J..

[7] Singhasivanon O.U., Lawpoolsri S., Mungthin M., Yimsamran S., Soonthornworasiri N., Krudsood S. (2019). Prevalence and alternative treatment of head-lice infestation in rural Thailand: A community-based study. Korean J. Parasitol..

[8] Kitvatanachai S., Kritsiriwutthinan K., Taylor A., Rhongbutsri P. (2023). Head lice infestation in pre-high school girls, Lak Hok suburban area, Pathum Thani province, in central Thailand. J. Parasitol. Res..

[9] Dudziński Ł., Kubiak T., Weiner M., Czyżewski Ł. (2024). Epidemiology of lice among Polish youth—5-year follow-up based on sales data in stationary pharmacies. Med. Res. J..

[10] Padzik M., Olędzka G., Gromala-Milaniuk A., Kopeć E., Hendiger E.B. (2023). The impact of the COVID-19 pandemic on the prevalence of head lice infestation among children attending schools and kindergartens in Poland. J. Clin. Med..

[11] Galassi F., Ortega-Insaurralde I., Adjemian V., Gonzalez-Audino P., Picollo M.I., Toloza C.A. (2021). Head lice were also affected by COVID-19: A decrease on Pediculosis infestation during lockdown in Buenos Aires. Parasitol. Res..

[12] Mumcuoglu K.Y., Hoffman T., Schwartz E. (2022). Head louse infestations before and during the COVID-19 epidemic in Israel. Acta Trop..

[13] Hatam-Nahavandi K., Ahmadpour E., Pashazadeh F., Dezhkam A., Zarean M., Rafiei-Sefiddashti R., Salimi-Khorashad A., Hosseini-Teshnizi S., Hazratian T., Otranto D. (2020). *Pediculosis capitis* among school-age students worldwide as an emerging public health concern: A systematic review and meta-analysis of past five decades. Parasitol. Res..

[14] Mumcuoglu K.Y., Pollack R.J., Reed D.L., Barker S.C., Gordon S., Toloza A.C., Picollo M.I., Taylan-Ozkan A., Chosidow O., Habedank B. (2021). International recommendations for an effective control of head louse infestations. Int. J. Dermatol..

[15] Gratz N.G., World Health Organization (1997). Human Lice: Their Prevalence, Control and Resistance to Insecticides: A Review 1985–1997.

[16] Burgess I. (1998). Head lice–developing a practical approach. Practitioner.

[17] Delie A.M., Melese M., Limenh L.W. (2024). Prevalence and associated factors of head lice infestation among primary school children in low- and middle-income countries: Systematic review and meta-analysis. BMC Public Health.

[18] Bartosik K., Buczek A., Zając Z., Kulisz J. (2015). Head pediculosis in schoolchildren in the eastern region of the European Union. Ann. Agric. Environ. Med..

[19] Tytuła A., Bartosik K., Jasztal-Kniażuk A., Buczek W., Błaszkiewicz A., Borzęcka-Sapko A. (2019). Analysis of the prevalence of pediculosis and scabies in orphanages and refugee shelters in south-eastern Poland. J. Educ. Health Sport.

[20] Bartosik K., Janczaruk M., Zając Z., Sędzikowska A., Kulisz J., Woźniak A., Jasztal-Kniażuk A., Kulbaka E., Tytuła A. (2022). Head Lice Infestation in Schoolchildren, in Poland—Is There a Chance for Change?. J. Clin. Med..

[21] Buczek A., Kawa I.M., Markowska-Gosik D., Widomska D. (2001). Pediculosis in rural schools of Lublin Province. Wiad. Parazytol..

[22] Buczek A., Markowska-Gosik D., Widomska D., Kawa I.M. (2004). *Pediculosis capitis* among schoolchildren in urban and rural areas of eastern Poland. Eur. J. Epidemiol..

[23] Sepehri M., Jafari Z. (2024). Prevalence and associated factors of head lice (*Pediculosis capitis*) among primary school students in Varzaqan Villages, Northwest of Iran. Zahedan J. Res. Med. Sci..

[24] Wungouw H., Memah V., Salaki C., Tarore D., Ottay R., Doda D., Rumampuk I., Rumampuk H. (2020). Prevalence of *Pediculosis capitis* and associated factors among primary school children at Kawiley Village, North Sulawesi, Indonesia. Sch. J. Appl. Med. Sci..

[25] Sayyadi M., Vahabi A., Sayyad S., Haji Sahne S. (2014). Prevalence of Head Louse (*Pediculus humanus capitis*) Infestation and Associated Factors Among Primary Schoolchildren in Bayengan City, West of Iran. Life Sci. J..

[26] Dagne H., Biya A.A., Tirfie A. (2019). Prevalence of *Pediculosis capitis* and associated factors among schoolchildren in Woreta town, northwest Ethiopia. BMC Res. Notes.

